# Linking atmospheric pollution to cryospheric change in the Third Pole region: current progress and future prospects

**DOI:** 10.1093/nsr/nwz031

**Published:** 2019-03-06

**Authors:** Shichang Kang, Qianggong Zhang, Yun Qian, Zhenming Ji, Chaoliu Li, Zhiyuan Cong, Yulan Zhang, Junming Guo, Wentao Du, Jie Huang, Qinglong You, Arnico K Panday, Maheswar Rupakheti, Deliang Chen, Örjan Gustafsson, Mark H Thiemens, Dahe Qin

**Affiliations:** 1 State Key Laboratory of Cryosphere Science, Northwest Institute of Eco-Environment and Resources, Chinese Academy of Sciences (CAS), Lanzhou 730000, China; 2 CAS Center for Excellence in Tibetan Plateau Earth Sciences, Beijing 100101, China; 3 Key Laboratory of Tibetan Environment Changes and Land Surface Processes, Institute of Tibetan Plateau Research, CAS, Beijing 100101, China; 4 Pacific Northwest National Laboratory (PNNL), Richland WA 99352, USA; 5 School of Atmospheric Sciences, Guangdong Province Key Laboratory for Climate Change and Natural Disaster Studies, Sun Yat-sen University, Guangzhou 510275, China; 6 Key Laboratory of Meteorological Disaster, Ministry of Education (KLME), Nanjing University of Information Science and Technology (NUIST), Nanjing 210044, China; 7 University of Chinese Academy of Sciences, Beijing 100049, China; 8 International Centre for Integrated Mountain Development (ICIMOD), Kathmandu G. P. O. 3226, Nepal; 9 Institute for Advanced Sustainability Studies (IASS), Potsdam 14467, Germany; 10 Department of Earth Sciences, University of Gothenburg, Gothenburg SE-405 30, Sweden; 11 Department of Environmental Science and Analytical Chemistry, The Bolin Centre for Climate Research, Stockholm University, Stockholm 10691, Sweden; 12 Department of Chemistry and Biochemistry, University of California San Diego, La Jolla CA 92093, USA

**Keywords:** atmospheric pollution, cryosphere, climate change, the Third Pole, coordinated monitoring network

## Abstract

The Tibetan Plateau and its surroundings are known as the Third Pole (TP). This region is noted for its high rates of glacier melt and the associated hydrological shifts that affect water supplies in Asia. Atmospheric pollutants contribute to climatic and cryospheric changes through their effects on solar radiation and the albedos of snow and ice surfaces; moreover, the behavior and fates within the cryosphere and environmental impacts of environmental pollutants are topics of increasing concern. In this review, we introduce a coordinated monitoring and research framework and network to link atmospheric pollution and cryospheric changes (APCC) within the TP region. We then provide an up-to-date summary of progress and achievements related to the APCC research framework, including aspects of atmospheric pollution's composition and concentration, spatial and temporal variations, trans-boundary transport pathways and mechanisms, and effects on the warming of atmosphere and changing in Indian monsoon, as well as melting of glacier and snow cover. We highlight that exogenous air pollutants can enter into the TP’s environments and cause great impacts on regional climatic and environmental changes. At last, we propose future research priorities and map out an extended program at the global scale. The ongoing monitoring activities and research facilitate comprehensive studies of atmosphere–cryosphere interactions, represent one of China's key research expeditions to the TP and the polar regions and contribute to the global perspective of earth system science.

## INTRODUCTION

The cryosphere (i.e. the portions of the Earth's surface where frozen water is found, including glaciers, ice sheets, snowpack, permafrost, sea ice and ice shelves) has changed rapidly over the last several decades [1,2]. Observations show that (i) the extent of Arctic sea ice has decreased [3,4], (ii) alpine glaciers have retreated at unprecedented rates [5], (iii) the duration of snow cover has decreased within high mountainous areas [6], (iv) permafrost has degenerated [7,8] and (v) the ice cover of lakes and rivers has frozen later and melted earlier [9]. These changes are mainly linked to climate changes induced by natural variability and human activity at both regional and global scales. In addition, atmospheric pollutants, especially those referred to as light-absorbing aerosols, such as black carbon (BC), brown carbon (BrC) and dust, can warm the atmosphere (e.g. [10,11]). After being deposited onto glacier ice or snowpack, these particles are considered to be light-absorbing impurities (LAIs). They can significantly reduce the surface albedos of glacier and snowpack and promote their melting [12–16]. The climatic effects of atmospheric pollutants and their roles in driving cryospheric changes, as well as the fate of atmospheric pollutants within the cryosphere and the associated environmental implications, are of great concern in the context of global warming [17]. It has been suggested that atmospheric pollution and cryospheric changes (APCC) are closely related and should be investigated in a coupled and integrated framework [18,19].

The Himalayas and the Tibetan Plateau are known as the ‘Third Pole’ and are hereafter referred to as the TP; they represent one of the most important cryospheric regions in the world [20,21]. The TP region includes the largest concentration of alpine glaciers in mid-latitude and vast areas of high-altitude permafrost and snow cover. Therefore, the TP region is of particular interest in studies of melting of the cryosphere and the associated changes in hydrological cycles, which affect the water supplies of over 1.4 billion people in Asia [22,23] and contribute to global sea-level rise [24–26]. Understanding cryospheric changes and the associated complex interactions with other components of the Earth system within the TP region is therefore key in designing adaptation measures to secure the livelihoods of local and regional human communities.

In this paper, we briefly review existing information on atmospheric pollution and its effect on cryospheric changes within the TP region. We then introduce the coordinated network for monitoring APCC over the TP region and review the research progress that depends on or is related to the network and the relevant recent achievements. Finally, we discuss future research priorities aimed at addressing the overarching science questions raised here and propose a path towards broadening the APCC network to the global scale.

## BACKGROUND INFORMATION ON CRYOSPHERIC AND ATMOSPHERIC CHANGES OVER THE TP

The main body of the TP region covers an area of over 2.5 × 10^6^ km^2^ and has an average elevation of ∼4000 m above sea level; it represents the single largest and highest-elevation landform feature on Earth [27]. The climate of the TP region is primarily influenced by alternating predominance of the westerlies in winter and the Asian monsoon in summer. In turn, the TP exerts profound thermal and dynamic effects on atmospheric circulation, thereby affecting the climate of Asia, the Northern Hemisphere and beyond [28,29]. The TP region hosts the largest mass of glaciers outside the polar regions [30] and includes extensive snow cover, alpine permafrost and lake ice; it is thus one of the important cryospheric reservoirs at the mid and low altitudes [20,21]. Due to its high elevation and the abundance of ice there, the TP region contains the headwater of many major Asian rivers; it provides water to over 1.4 billion people who live downstream and is therefore known as the ‘Water Tower of Asia’ [22]. However, during the last few decades, the TP has experienced significant and rapid climate warming [31,32], accompanied by intensive interactions among the atmosphere, hydrosphere, cryosphere and biosphere that represent diverse land-surface processes.

Glaciers are among the most studied cryospheric elements in the TP region. The changes in glaciers within the TP region display spatially heterogeneous trends. The greatest degree of glacial recession is found in the Himalayas, but the degree of shrinkage decreases in the interior of the TP region, and the glaciers of the Karakorum and the eastern Pamirs are exceptionally stable or are advancing slightly [30,33,34]. Snow cover has decreased over the past two decades; a total reduction of 5.7% occurred from 1997 to 2012 [35] and the duration of seasonal ground freezing decreased, due to winter warming [36]. Permafrost temperatures increased by 0.2–0.5°C and its extent decreased by 100 000 km^2^ from the 1970s to 1990s within the hinterland areas of the TP region, leading to pervasive permafrost degradation ([31] and references therein). Lake-ice phenology, which is controlled by both climatic and local factors, varies among different regions; notably, a number of Tibetan lakes did not completely freeze up during some winter seasons between 2001 and 2010 [34,37]. Taken together, these observations reflect a changing and mostly diminishing cryosphere within the TP region. These changes generate concerns regarding hydrological processes, natural hazards and environmental changes, which could potentially affect the sustainability of the livelihoods and wellbeing of humans and eco-social sustainable development on the TP and in its surroundings [2].

While intense attention has been paid to cryospheric changes, little work has investigated atmospheric pollution over the TP region, which has long been regarded as remote and pristine. Records reconstructed from environmental archives, such as glacial ice cores and lake sediments, reveal distinguishable anthropogenic disturbances that are mainly associated with Asian emissions since the 1950s (e.g. [38–40]). From a global perspective, several available monitoring and comparative studies indicate that the atmospheric environment of the TP region mostly reflects global background conditions. For example, observed aerosol optical depth (AOD), which is frequently used as a simple indicator of air cleanliness, has typically been low and stable over time over the TP region, even at urban sites (levels of 0.1× have been measured at Haibei and Lhasa) [41]; moreover, the AOD measured at Nam Co station—a regional background site—is even lower (0.0× in 2006–07) [42].

**Figure 1.**
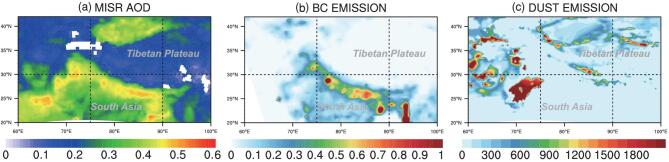
AOD (a), BC (b) and dust (c) emissions over the TP region and its surroundings from 2000 to 2009 (units: mg m^−2^ day) (based on and modified from [46–48]).

However, note that the TP is located between East Asia and South Asia—two regions with some of the world's most intensive anthropogenic emissions. The satellite-based AOD images clearly show strong contrasts between South Asia and the TP region (Fig. 1a). High levels of atmospheric pollution, known as ‘atmospheric brown clouds’ (ABC) [43], extend from South Asia and accumulate on the southern slope of the Himalayas, representing the overflow of the potential effects of atmospheric pollution from the southern Himalayas into the TP region. For example, large amounts of BC are found over the northern Indo-Gangetic Plain (Fig. 1b). In addition, the TP is also frequently influenced by dust events (e.g. [44]) and the dust emissions from its surroundings and the plateau itself are comparably high (Fig. 1c).

Studies have indicated that atmospheric pollution generated in South Asia can be transported via the Indian monsoon and westerlies, and thus reaches the hinterlands of the TP region [45]. These atmospheric pollutants are subsequently deposited at high altitudes, leading to environmental effects. Nonetheless, most studies are based on episodic monitoring and investigation at widely dispersed sites or in specific regions; few data from systematic long-term monitoring programs are available. The sampling and measurements reported by individual studies have been performed with different instruments and techniques, resulting in inconsistencies and uncertainties in the intercomparison of these data and regional modeling. Furthermore, studies are usually limited to one homogenous atmospheric or cryospheric field, and atmospheric pollutants and their linkages to their counterparts in the cryosphere are poorly understood. Therefore, integrated study is critical for assessing the climatic and hydrological effects of atmospheric pollutants in the atmosphere, as well as in glaciers and snowpack.

## A COORDINATED NETWORK DEDICATED TO MONITORING APCC OVER THE TP REGION

**Figure 2.**
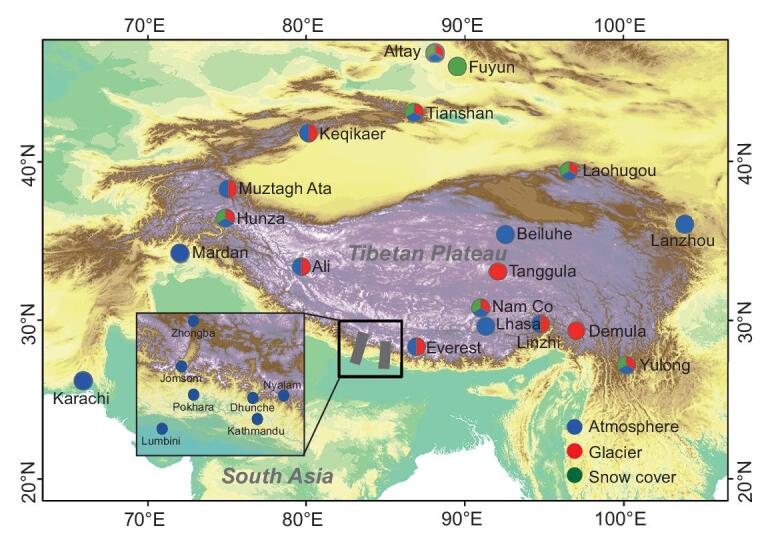
Sites where atmospheric pollution and cryospheric changes are monitored in the TP region and beyond.

In 2013, we initiated a coordinated APCC monitoring network with the overarching goal of performing more integrated and in-depth investigations of the origins and distributions of atmospheric pollutants and their impacts on cryospheric changes over the TP region (Fig. 2). The specific goals of this network are to:

- characterize the chemical compositions and levels of atmospheric pollutants and depict their spatial and seasonal variation over the TP region;
- identify the sources of atmospheric pollutants and reveal the pathways and mechanisms by which trans-boundary atmospheric pollution is transported to the TP region; and
- investigate the role of atmospheric pollutants deposited as LAIs in the melting of glacier ice and snow cover and, further, quantify the contribution of LAIs to glacier and snowpack melting, and determine the fates of environmentally relevant pollutants within glaciers and snowpack and their scavenging processes during the melting of snow and ice.

The prototype monitoring network includes seven sites distributed along a south–north transect across the central Himalayas (small panel in Fig. 2) and two other sites (Lhasa and Nam Co) as references of the inland TP in early 2013. The construction and operation of all the sites are jointly supported by the Chinese Academy of Sciences (CAS), the International Center for Integrated Mountain Development (ICIMOD) and the Sustainable Atmosphere for the Kathmandu Valley (SusKat) project (http://www.iass-potsdam.de/en/research/air-quality/suskat), as well as contributions from many scientists and local residents. Additional sites are gradually launched as the existing monitoring network is maintained, and this network covers the large spatial extent of the TP region and areas beyond (Fig. 2).

The observational and sampled quantities are summarized in Table 1. The observation of atmospheric pollution is generally composed of real-time monitoring and discrete aerosol filter sampling. Real-time monitoring is performed at several sites (e.g. Kathmandu, Nam Co and Everest), where sufficient logistical support and manpower are available. To perform the filter sampling, we run a total suspended particle (TSP <100 μm) auto-sampler for 24–48 hours every 6 days. During the pre-monsoon season, which generally extends from March to May and features the breakout of ABCs, the sampling frequency is increased to every 2–3 days. The filters are punched into small pieces to permit analysis of multiple chemical parameters, including BC, organic carbon (OC), insoluble ions and heavy metals. Geochemical parameters that permit robust apportionment of pollution sources, such as the isotopes of carbon and the abundances of various elements (e.g. lead and mercury) and organic molecular tracers (e.g. anhydrosugars and resin acids), are measured for selected filters. Measurement protocols that are similar to those applied to the filters are applied to the snow and ice samples obtained from glaciers and snow cover.

**Table 1.** Observational parameters, instrumentation and temporal resolution of the APCC monitoring network in the TP region.

Research content	Sampling/observational parameters	Instrumentation	Frequency
Online measurements	Aerosol optical properties	CIMEL Sunphotometer	Every 15 mins
	Aerosol concentrations: PM_2.5_, PM_10_	Thermo RP1400	Real-time
	Gaseous precursors: SO_2_, NO_x_, CO, O_3_; Toxic gases (atmospheric mercury)	Thermo 42I, 43I, 45I, 49I analyser; Tekran 2537	Hourly
	BC	Aethalometer AE33	Every 5 mins
Filters	Total suspend particles: EC/OC, brown carbon (BrC), inorganic ions, trace element, and isotopes and organic tracers	TSP sampler	3–6 days
Glaciers and snow cover	Snow and ice samples: dust, EC/OC, BrC, WSOC, inorganic ions, trace elements and isotopes and organic tracers		1–2 times per year
	*In situ* albedo	ASD Handheld 2-spectroradiometer	

In observing and collecting samples from the cryosphere, benchmark glaciers and snow-covered regions are chosen for meteorological and mass balance measurements and periodic sampling to investigate the preserved atmospheric pollutants. Special emphasis is placed on pollutants with climatic and health effects, including LAIs, heavy metals and persistent organic pollutants. *In situ* albedo measurements are conducted using a portable spectrometer.

The monitoring data and measurements of samples can be integrated into models and used for validation. These practices provide insight into how atmospheric pollution wafts over the Himalayas and the effects it has on the glaciers of the region. Coupled climate–atmospheric chemistry models (e.g. WRF-Chem) are used to investigate the transport and contributions of trans-boundary atmospheric pollution to the TP region. A regional climate model (RegCM) is also used to simulate the climate effects of atmospheric pollution at a regional scale. The SNow ICe Aerosol Radiative (SNICAR) model coupled with a distributed hydrologic model is used to diagnose the contributions of LAIs to snow and ice melting. All of the model results are evaluated and serve in turn as guidance for adjusting the field monitoring and sampling strategies, such as the spatial resolution and temporal frequency of measurements.

In summary, we seek to obtain a continuous observational dataset and systematic sampling of the atmospheric and cryosphere components from cross-sections of the TP region to improve our understanding of the distributions, variations in and transport of atmospheric pollutants and to assess their impacts on cryospheric changes over the TP region.

## IMPROVED UNDERSTANDING OF APCC IN THE TP REGION

The APCC in the TP region has been the subject of increasing interest in recent years. For example, Qian *et al.* [15] summarized various technical methods of measuring LAIs in snow and ice and reviewed the progress in data measurement and modeling in high-latitude and high-altitude regions; they emphasized that the TP region is one of the hottest spots for investigating the climate impacts of LAIs in snow and ice, but the lack of LAI measurement data has impeded robust estimation of the related radiative forcing. Later, Gertler *et al.* [18] reviewed observational data of BC in the atmosphere and in snow and ice in the Himalayas; they showed the systematic differences of BC concentrations between observation and models, and underlined the importance of consistent measurement protocols to facilitate model verification. Here, we provide an up-to-date synthesis of existing knowledge that helps to enhance the links between APCC over the TP region; most of the observational data are from APCC projects with consistent monitoring and measurement protocols.

### Atmospheric pollution: composition, variation and sources

**Figure 3.**
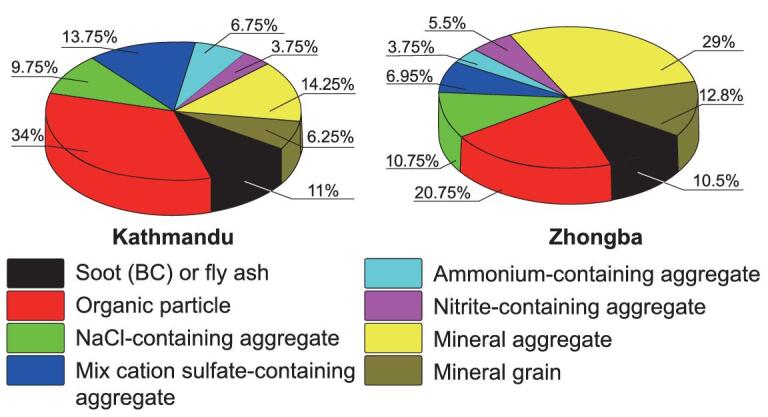
The composition of aerosols at sites in the southern and northern Himalayas.

Quantification of the compositions of atmospheric particles is important in increasing our knowledge and understanding of their sources and their radiative forcing effects in the atmosphere. Studies on individual particles show that high concentrations of pollutants are found at urban sites (e.g. BC for Kathmandu) and high portions of natural crustal dust are observed at remote sites (e.g. Zhongba) [49] (Fig. 3). Meanwhile, high observed IndP/(IndP + BghiP) and Fla/(Fla + Pyr) ratios suggest that atmospheric polycyclic aromatic hydrocarbons (PAHs) from both urban and rural sites are mainly produced by biomass burning, followed by coal burning and petroleum combustion [50]. This conclusion is also confirmed by high levoglucosan/mannosan and syringic acid/vanillic acid ratios at the typical rural site of Lumbini in South Asia [51]. Due to the combined effects of biomass combustion and high secondary organic carbon production, the measured OC/BC ratios are high (greater than 6) in most remote areas of the TP region [52–55]; these values are similar to some areas in Europe [56] but are higher than those of typical urban areas in the TP region [57–59].

The temporal and spatial variations in atmospheric pollution over the TP region have been further illustrated. Generally, the concentrations of air pollutants over the TP region are much lower compared with its surrounding areas, especially within the inner part of the TP region [60]. For instance, the annual mean BC concentration at Nam Co station is 74 ng m^−3^ [61], whereas air pollution is serious in urban areas. For example, measurements of carbonaceous aerosols [51,58,62,63], PAHs [64,65], heavy metal elements [66–68] and precipitation chemistry [58,68] show serious pollution of the atmosphere by local emission sources in the urban areas on the northern and southern slopes of the Himalayas. The spatial distributions of BC and particle-bound PAHs also suggest clear decreases in loading along the south–north valleys of the Himalayas [49,50,69], mainly due to the relative proximity of these sites to the emission sources of South Asia and the depositional fractionation of pollutants during their transport up-valley. Fossil contributions of BC decrease from 49% at Lumbini on the India-Ganges Plain (IGP) to 30% at Zhongba in the TP region [69]. The degree of atmospheric pollution on both sides of the Himalayas shows clear seasonal variations, and it generally reaches its maximum and minimum in the non-monsoon and monsoon seasons, respectively [51,52,65]. This temporal distribution coincides with the ABC outbreaks and heavy precipitation that occur during these seasons.

Increasing evidence suggests that, besides BC, some components of OC also absorb sunlight, in addition to their scattering effects. Therefore, it is very important to study the *in situ* measured light-absorption characteristics of OC to better understand the radiative forcing effects of carbonaceous particles. Our research reveals that the mass absorption cross-sections of the water-soluble organic carbon (WSOC) of carbonaceous particles at 365 nm at two remote stations (Linzhi and Everest) are 0.84 ± 0.40 and 1.18 ± 0.64 m^2^ g^−1^, respectively; these values are significantly higher than the corresponding values for dissolved organic carbon (DOC) in precipitation. This difference is mainly due to the high proportion of gaseous components with low light-absorption ability in precipitation DOC [53]. In addition, MAC_WSOC_ displays obvious seasonal variations; high and low values appear in winter and summer, respectively, reflecting the photobleaching of the light-absorbing components of WSOC caused by fluctuations in sunlight intensity. Correspondingly, the MAC_EC_ values at 632 nm for the aerosols observed at the two stations mentioned above are 6.85 ± 1.39 and 6.49 ± 2.81 m^2^ g^−1^, respectively; these values are similar to those of freshly emitted uncoated particles (6.5 ± 1.05) [70]. This result indicates that the enhancement of MAC_EC_ is not significant, even though these particles may have experienced long-range transport from South Asia.

### Trans-Himalayan atmospheric pollution: evidence, transport pathways and mechanisms

Accumulated atmospheric pollution at the southern foot of the Himalayas can traverse the Himalayas and reach the inland TP region. Episodic cross-Himalayan pollution is transported through the major south–north valleys and becomes lofted over the Himalayas. For example, ground- and satellite-based remote sensing data indicate that a severe aerosol pollution episode occurred in this region in March 2009. Specifically, during the days prior to this event, the winds over the IGP were generally weak at lower levels, permitting the accumulation of pollutants and thus the formation of ABCs. The subsequent passage of synoptic-scale troughs led to southwesterly flow in the middle troposphere over northern and central India, carrying the polluted air masses across the Himalayas [71].

Local meteorological processes, such as mountain peak-valley wind systems, may also facilitate the trans-Himalayan transport of atmospheric pollutants [52,71,72]. On the southern slope of the Himalayas, the daytime up-valley breezes deliver air pollutants from the foothills to higher altitudes (>5000 m a.s.l. (above sea level)). In contrast, down-valley winds are dominant on the north side in the daytime and these winds peak in the afternoon. The intense daytime valley wind-circulation pattern in this region makes the valleys efficient channels for the transport of air pollutants across the Himalayas [72]. On a larger spatial scale, recent modeling work using RegCM4.3 provides further characterization of the sources and transport pathways of carbonaceous aerosols over the TP region. This work confirms that the westerly winds that prevail throughout the year can transport carbonaceous particles and dust from central Asia to the northern TP. Meanwhile, during monsoons, aerosols can cross the Himalayas and be transported to high altitudes by the southwesterly winds over South Asia [47].

Based on the modeling assessment [73], the majority of the BC found in the TP region is transported from South Asia; this source contributes to 40–80% (mean of 61.3%) of the surface BC in the non-monsoon season and 10–50% (mean of 19.4%) during the monsoon season. In the northeastern TP, BC from eastern China accounts for less than 10% of the total in the non-monsoon season, but up to 50% during the monsoon season.

### The deposition of atmospheric pollution into the cryosphere: distribution and impacts

The deposition of LAIs on glaciers and snow cover reduces the albedos of ice and snow and accelerates their melting, due to positive feedbacks [10,74]. LAI concentrations can differ by 1–2 orders of magnitude among different glacier surface types (e.g. ice, fresh snow, aged snow and granular ice) due to topographic effects, local/regional emissions and/or post-depositional effects (e.g. [75,76–83]). Geographically, higher concentrations are more likely to occur in the marginal areas than farther inland within the TP region [76]. Further, the LAI concentrations in aged snow and granular ice are found to be much higher than those measured in fresh snow and glacier snow pits. For instance, in the Pamir region, the concentrations of BC and dust in aged snow can reach 1621 ng g^−1^ and 334.10 μg g^−1^, respectively; however, in fresh snow, these concentrations are 95 ng g^−1^ and 2.20 μg g^−1^, respectively [80]. In the central TP, the average concentrations of OC, BC and dust in aged snow are 611 ± 468 ng g^−1^, 247 ± 119 ng g^−1^ and 39.4 ± 24.3 μg g^−1^, respectively, whereas the average concentrations of OC, BC and dust in fresh snow are 158 ± 42 ng g^−1^, 42 ± 6 ng g^−1^ and 1.89 ± 0.92 μg g^−1^, respectively [84]. In the southern TP, the average concentrations of OC, BC and dust in aged snow are approximately 3913 ± 5235 ng g^−1^, 1550 ± 2117 ng g^−1^ and 497 ± 712 μg g^−1^, respectively, and, in fresh snow, the average concentrations of these substances are 250 ± 70 ng g^−1^, 57 ± 26 ng g^−1^ and 25 ± 8.96 μg g^−1^ [83].

**Figure 4.**
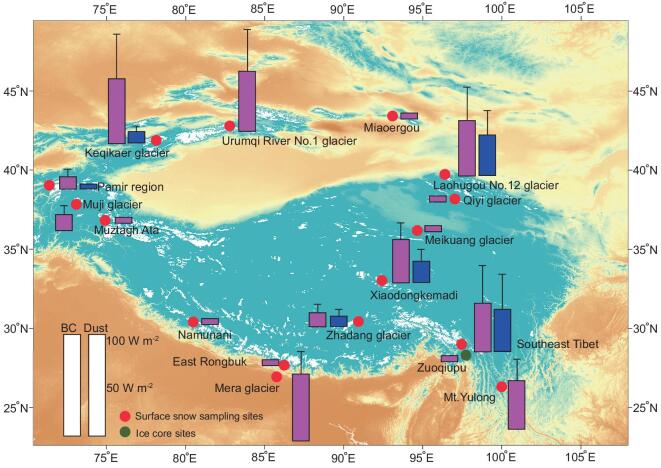
Effects of BC and dust on radiative forcing over the glaciers of TP.

On average, the BC and dust found in the snow and ice on the surfaces of glaciers are responsible for approximately 20% of the albedo reduction within the TP region. The instantaneous radiative forcing induced by BC and MD in fresh snow is estimated to be approximately 4.78 ± 1.27 W m^–2^ in the southeastern TP and 7.09 ± 2.10 W m^–2^ in the central TP [83]. Meanwhile, the instantaneous radiative forcing for aged snow or granular ice can reach 100 W m^–2^ [78–80,83,85] (Fig. 4). The BC concentrations and the associated annual mean radiative forcing have increased by a factor of three over the last several decades, as reconstructed from ice cores collected from glaciers in the southern TP [81,86]. In addition, the effects of LAIs on albedo reduction and radiative forcing can lead to approximately 15% of the total glacier melt in the southeastern TP [83] and summer melt-rate increases of up to 6.3% (7 cm a^–1^) on the glaciers in the Pamirs [80]. Compared to the melting rate on the Mera glacier in the Himalayas, the combined contributions of dust and BC to surface melting can reach a maximum value of 26% [87].

The darkening effects of biological impurities (e.g. algae and cyanobacterial cells that aggregate or attached on mineral fragments) on snow have also been acknowledged as causing important reductions in the surface albedo of glaciers, known as ‘bioalbedo’ [88,89]. Due to the abundance of microbial communities and high DOC/OC ratios in the TP region [90,91], understanding how biological and DOC affect snow melting is urgent in order to quantify how the glacier melting will evolve in the future.

### Climatic effects of atmospheric aerosols

Some previous studies have used general circulation models (GCMs) to simulate aerosol transport and climatic effects over the TP region [92–94]. However, given the coarse resolutions used in GCMs, it is difficult, if not impossible, to capture the effects of terrain on climatology [95] and such models perform relatively poorly over the TP region [17]. RegCMs with high resolutions address the shortcomings of GCMs with coarser grids, and some updated RegCMs include treatments of atmospheric chemistry [96] and snow/ice-aerosol radiative feedback packages [46]. These models have begun to be widely used in modeling aerosols and their climatic effects over the TP region.

Using a coupled RegCM, Ji *et al.* [48] showed that mixed anthropogenic aerosols induce a delay in the onset of the summer monsoon of one to two pentads in central and southeast India, whereas this onset advances by one to two pentads in northeastern India and Myanmar. Using an RegCMs coupled with an aerosol module, Nair *et al.* [97] demonstrated that the coupled model accurately represents the annual, seasonal and diurnal variations in anthropogenic aerosols over the Himalayas and South Asia. Due to the heavy pollution derived from emissions of carbonaceous aerosols, Ji *et al.* [47] simulated the spatial distribution of carbonaceous (BC and OC) aerosols over the TP region; they found relatively high concentrations in the southern TP, consistently with ground-based observation. In the southern TP, carbonaceous aerosols can warm (cool) the surface air by 0.1–0.5°C in the monsoon (non-monsoon) season and BC wet deposition is significantly enhanced during the monsoon season compared to the non-monsoon season.

The diffusion mechanism of atmospheric pollution is also revealed using the WRF-CHEM model [98]. In Southern Asia, the heating of the middle to lower troposphere induced by BC and BrC leads to increases in the stability of the atmospheric boundary layer and weaker diffusion of meteorological conditions, resulting in further heavy pollution. However, due to the low aerosol concentration over the TP region, the heating effects of BC and BrC are insufficient to cause variations in the thermal structure in the boundary layer. This result implies that air pollutants spread quickly through convection, transport and deposition once transported over the TP region.

In addition to the climatic effects of aerosols effects in the atmosphere, the effects of the light-absorbing particles deposited on snow and ice have also been investigated using GCMs and RegCMs. Using a high-resolution GCM with an observationally constrained BC aerosol forcing, Xu *et al.* [99] demonstrated that increases in atmospheric CO_2_ and BC concentrations at the TP altitudes exerted a warming of 1.7°C and 1.3°C, respectively; therefore, BC is an important contributor to the snow-retreat trends over the TP region. Equilibrium GCM experiments designed by Qian *et al.* [17] showed that the efficacy of BC-in-snow in inducing snowmelt is one to three times greater for the snow-cover fraction and two to four times greater for the snow water equivalent (SWE) than that of CO_2_. Using an RegCM, Ji *et al.* [100] showed that dust-in-snow induces a warming of 0.1–0.5°C and SWE decreases of 5–25 mm over the western TP and the Pamir and Kunlun Mountains. Meanwhile, BC-in-snow results in a warming of 0.1–1.5°C in the western TP and the Himalayas, and the loss of SWE through melting exceeds 25 mm in some parts of the regions mentioned above [46].

Taken together, atmospheric aerosols exert significant impact on both the warming and the cooling of the atmosphere and the duration of snow cover.

### Accelerated melting of the cryosphere, the related eco-environmental effects and beyond

The light-absorbing portion of atmospheric pollution and the deposition of atmospheric pollutants onto snow and ice surfaces contribute to increasing losses of snow and ice and cause accelerated melting of the cryosphere, particularly in the context of ongoing warming. The most notable eco-environmental effect of the melting of the cryosphere is its impact on water availability and consequently hydropower generation, agriculture and cities. Additionally, some of the atmospheric pollutants deposited on snow and ice surfaces and preserved in the cryosphere can undergo complex physical, chemical and biological interactions. As such, their transformation and fate, together with the melting of the cryosphere, have attracted increasing amounts of attention, given the eco-environmental effects of these pollutants on the receiving environments. Accumulations of pollutants are often found in dust-enriched snow strata, superimposed ice layers and cryoconite, usually at concentrations that are several times or orders of magnitude greater than their levels in glacier ice and fresh snow. These high levels of pollutants reflect the effects of percolation and melting and the related processes of migration within snow and ice, transformation and re-aggregation. For example, mercury is consistently found at elevated levels in visible dust layers in snowpits excavated on glaciers in the TP [101,102]. Additionally, mercury levels in superimposed ice layers reach 306.5 ng L^−1^ on the Laohugou No. 12 glacier in the Qilian Mountains [103]. The close correlation between insoluble particles and mercury has been reproducibly confirmed in a series of recent studies performed in different parts of the TP region [104]. Cryoconites, which consists mainly of dust-sized mineral grains, has been found to readily accumulate anthropogenic pollutants and contains extraordinarily high levels of BC and trace elements [105]. All of these results illustrate the heterogeneity of pollutants in glaciers.

The cryosphere represents a temporary reservoir for pollutants; therefore, melting of the cryosphere discharges these stored pollutants into the meltwater that drains into glacier-fed river basins and flows into the downstream environments. Trace elements in glacier-fed rivers generally show clear diurnal variations with peaks related to periods of intense glacier melting [106–108]; these results indicate substantial exports of pollutants by melting glaciers. Ultimately, melting glaciers may release pollutants via both direct pathways (i.e. the melting snow and ice) and the hydrodynamic remobilization of glacier-fed watercourses [108]. Further, the latter effect may be more effective, given the steepness of glacier-fed rivers, which can enhance their dynamics. A case study performed in a typical glacier basin in the inland portion of the TP region revealed higher mercury yields compared to those in polar regions [108]. The nature of melting glaciers as sources of legacy pollutants, which had previously been noted in the high Canadian Arctic and the Alps (e.g. [109,110]) has been confirmed by profiles of persistent organic pollutants (POPs) measured in proglacial alluvial deposits in the central TP [111], as well as in a survey of POPs in glacier-fed rivers originating in the western Himalayas [112]. The potential eco-environmental risks of the release of pollutants from melting glaciers, although not extensively and quantitatively studied within the TP region [113], will likely be enhanced in the context of climate change; this process may represent a hidden hazard with latent and lasting effects on distant environments and societies [114]. Permafrost represents an even larger repository of chemicals, and its degradation and the accompanying mobilization of carbon and toxic pollutants into other components of the Earth system have significance for regional and global climatic and environmental changes [115].

**Figure 5.**
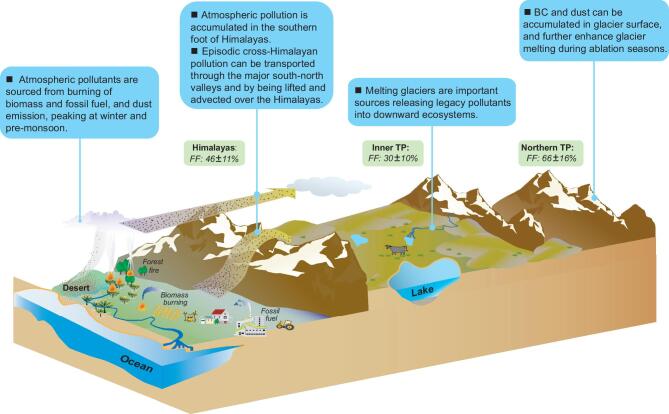
Illustration showing current understanding of APCC over the TP region. FF, fossil fuel fraction of BC in glacier snow [69].

In Fig. 5, we summarize this overarching discussion in terms of its highlights.

## FUTURE PROSPECTS

### Research priorities

The growing monitoring network and ongoing research represent a foundation that promotes improved understanding of atmospheric pollution and its impacts on cryospheric changes over the TP. We suggest a number of future research priorities to address the key scientific questions raised. These priorities are listed and explicated below.

#### Constraining the contribution of trans-boundary atmospheric pollution

Regarding this point, the following questions are of interest. To what extent do the trans-boundary and (from a broader perspective) exogenous atmospheric pollution contribute to the total loading of atmospheric pollution over the TP region? Moreover, what are the characteristics of the spatial coverage and seasonality of atmospheric pollution? Such questions can be answered by identifying robust chemical tracers that can be used to further characterize the sources of exogenous atmospheric pollution. Further, sophisticated experiments that couple ground-based monitoring data with models are needed to constrain the scale of exogenous atmospheric pollution over the TP region.

#### Measurements of pollutants in ambient air, glaciers and snowpack

The analysis of aerosol samples from the regular sampling performed as part of the monitoring network provide a general understanding of the distribution of pollutants. Nonetheless, the link between airborne pollutants and the portion deposited onto snow surfaces remains poorly understood. Concerted sampling and measurement of ambient air and snow/ice samples are needed in order to better understand the characteristics of the atmospheric pollutants that are deposited on glaciers and preserved in glacier ice. The roles of BC, dust and other presently unidentified substances and processes in promoting glacier melt within the TP region should be investigated. Mountain glaciers feature highly heterogeneous surfaces that transition from snow to firn, ice and ice mixtures and thereafter morainic material a short distance downwards. Therefore, it is critical to perform extensive sampling and measurements covering entire glaciers, namely in both the accumulation and ablation zones, to quantify the contributions of LAIs to glacier melting. An effective glacier albedo model should be validated to better simulate the LAI-driven albedo changes on mountain glaciers within the TP region. This model could then be interactively coupled with a RegCM to assess the impacts of atmospheric pollution and climate change on changes in glacier and snowpack from a broader perspective.

#### Fate of pollutants in the cryosphere and their environmental significance

The fate of pollutants preserved in glaciers and their subsequent impact on downstream ecosystems in the TP region remain imperfectly understood. This issue is important, considering that the periods of intensive glacier melting and rapid biomass accumulation are in phase with one another and the efficient uptake of pollutants in the ecosystems downstream of the TP [116]. The distributions and transformation of toxic pollutants, such as heavy metals and POPs, within glacial snow and ice should be investigated, particularly in the glacier ablation zone. Coordinated sampling of glacier and meltwater-fed runoff should be conducted during the summer melt season to investigate the export and transport of pollutants from glaciers to downstream ecosystems. The export flux and the bioavailability of pollutants to the glacier-fed biota are preferential indices for assessing the environmental significance of pollutants released by glacier melt.

In addition to these scientific questions, we should promote the sharing and integration of data to facilitate the availability of these data and cooperative studies. Moreover, we should expedite the translation of research results into knowledge and practices to promote the sustainable development of the TP region.

### APCC—a ‘three poles’ view

APCC are increasingly interrelated, both within the TP region and in other polar and high mountain regions, such as the Alps and Andes, where similar concepts and projects are dedicated to achieving an integrated understanding of the links between these aspects [19]. The TP represents one of the best mid-latitude counterparts to the polar regions, and they receive primarily exogenous atmospheric pollution, but the climate regimes, landforms and major cryospheric elements and their properties are different. Therefore, consolidated investigations and comparison studies among these regions would be beneficial to illustrate the interaction of APCC for a global perspective.

**Figure 6.**
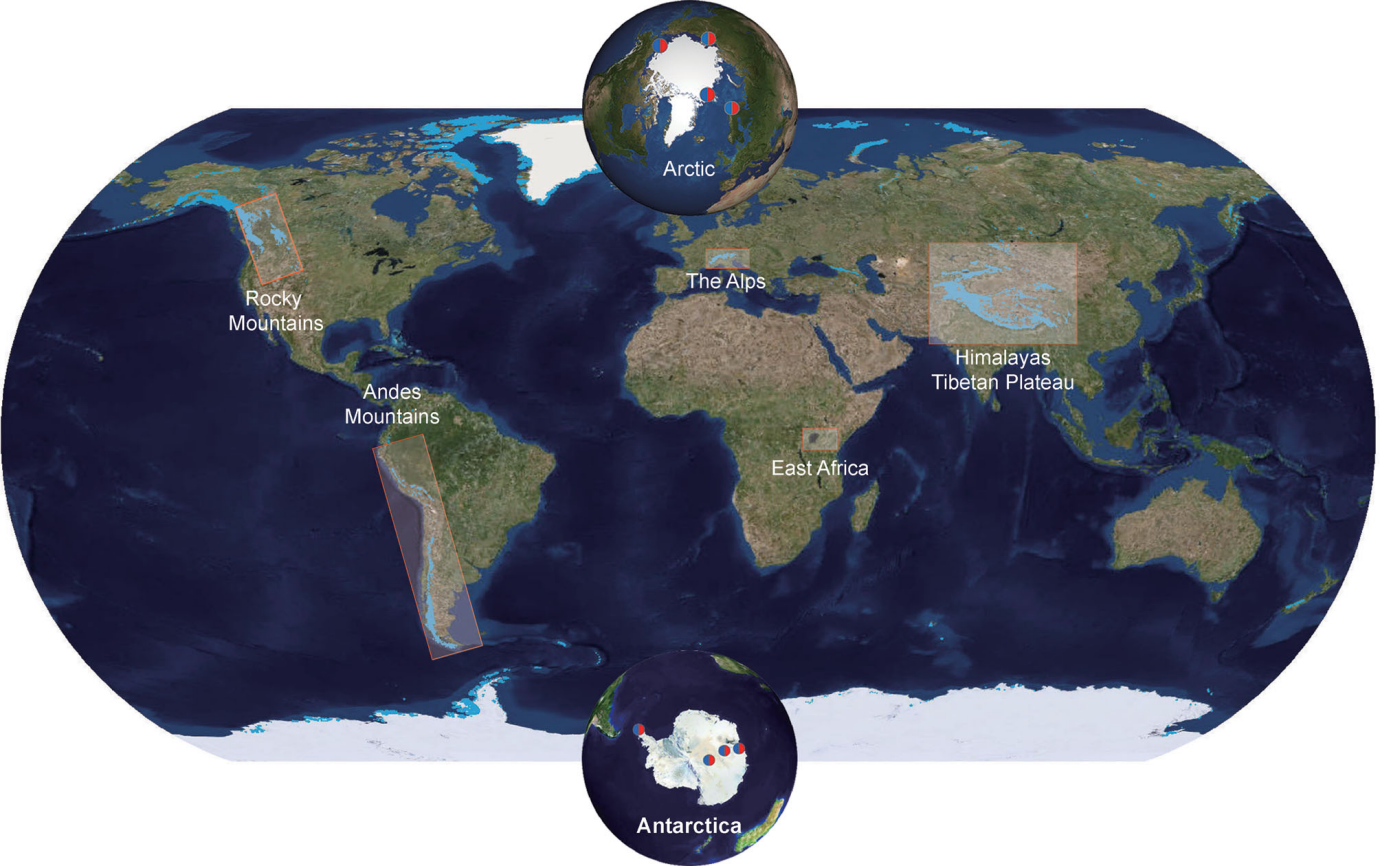
Schema of future research project for assessing atmospheric pollution and cryospheric changes at a global scale. Rectangular frames indicate key cryospheric regions at mid to low latitudes; light-blue dots denote glaciers included in the Randolph Glacier Inventory 5.0 (https://www.glims.org/RGI/randolph50.html); circles denote monitoring sites refer to legend in Fig. 2.

We propose a global framework of APCC to perform coordinated observations in the TP region, as well as in the Arctic and Antarctica (Fig. 6). With this, we aim to

- operate an expanding monitoring network continuously to obtain systematic samples and measurements of LAIs and toxic pollutants in the atmosphere and cryosphere, as well as other relevant parameters (e.g. meteorological indices, cryospheric variables and the *in situ* albedo of snowpack surfaces);
- depict the differences in composition, distribution and deposition of atmospheric pollutants among the three polar regions;
- quantify the relative importance of different atmospheric pollutants on cryospheric changes and assess potential feedback effects;
- conduct comparative research among different regions to help quantify the commonalities and differences in the underlying mechanisms by which atmospheric pollutants drive and respond to cryospheric changes over cryospheric regions globally.

In turn, all these studies are essential to provide scientific references for effective strategies that can reduce regional atmospheric pollution, thus improving the management of the water resources on which regional populations depend and mitigating global sea-level rise. The results from this project will also be valuable in advancing our understanding of the interactions among the different components of the Earth system.
